# An Evaluation of the Measurement Properties of the Five Cs Model of Positive Youth Development

**DOI:** 10.3389/fpsyg.2015.01941

**Published:** 2015-12-22

**Authors:** Ronan J. Conway, Caroline Heary, Michael J. Hogan

**Affiliations:** School of Psychology, National University of Ireland GalwayGalway, Ireland

**Keywords:** positive youth development, confirmatory factory analyses, measurement invariance, adolescence, gender development, Ireland

## Abstract

There is growing recognition of the need to develop acceptable measures of adolescent's positive attributes in diverse contexts. The current study evaluated the measurement properties of the Five Cs model of Positive Youth Development (PYD) scale (Lerner et al., 2005) using a sample of 672 Irish adolescents. Confirmatory factor analyses indicated that a five-factor model provided a good fit to the data. The internal reliability and construct validity of the Five Cs model were supported, with character the strongest predictor of contribution, while connection was the strongest predictor of risky-behaviors. Notably, confidence was significantly negatively related to contribution, and positively related to risky-behaviors. Multi-group hierarchical nested models supported measurement invariance across early- (11–14 years) and late- (15–19 years) adolescent age groups, with partial invariance found across gender. Younger adolescents evinced higher PYD, while PYD was associated with higher contribution and lower depression and risk-behaviors across all groups. The application of the PYD framework as a measure of positive functioning across adolescence is discussed.

## Introduction

The pervasive influence of the “deficit perspective” of youth is acknowledged to have shaped the twentieth century discourse of adolescent research, policy, and practice (e.g., Bowers et al., 2010). This discourse had as its focal point the measurement of risk and problem behaviors. However, a more recent approach to adolescent development has emerged that advocates for the strengths of youth, and espouses the positive qualities and desirable outcomes that parents, teachers, practitioners, and society wish to develop. This approach is referred to as the Positive Youth Development (PYD) perspective.

Several conceptualizations and theoretical frameworks of PYD have been conceived (for a review, see Lerner et al., 2009). A recent review of PYD frameworks has indicated that the Five Cs Model of PYD is the most empirically supported framework to date (Heck and Subramaniam, 2009). However, a number of concerns remain, including; concern about the indicators used to operationalize the Five Cs Model of PYD across adolescence; concern about the manifestation of PYD across gender; and concern about the generalizability of the Five Cs Model of PYD outside of North America. For instance, while there may be many commonalities in the forms of PYD across cultures, the developmental emergence and frequency of some forms of PYD may differ due to cultural and societal differences. Studies of the Five Cs Model of PYD in cultures other than North America are needed to further assess the development and structure of PYD.

Ireland provides a fitting context in which to measure PYD, as contextual factors such as economic instability, recession and a high level of youth unemployment highlight the pressing need to develop policy and practice to support young people using empirically robust measures. In particular, Ireland has one of the highest rates of young people not currently in education, employment or training (22%; Eurofound, 2012), high youth unemployment (26.3%; Eurostat, 2013) and worrying trends related to youth depression, alcohol consumption and suicide (UNICEF, 2011; Headstrong, 2012; National Office for Suicide Prevention, 2012). In contrast, a recent study found that 68% of a representative sample of Irish adolescents reported being happy with family life (Headstrong, 2012), while four out of five Irish adolescents report being happy (UNICEF, 2011). The broader Irish context also appears supportive of prosocial behavior, as figures suggest that Irish people spend more time per day volunteering compared to other industrialized countries (Better Life Index, 2013). In a cross-country analysis of young people's subjective well-being across 28 OECD countries, Ireland ranked an average 14th, in comparison to the US which ranked 27th (Bradshaw et al., 2013). Thus, research findings present a complex picture of the Irish youth development context. However, while existing indicators of community wellbeing suggest the Irish context in general may be conducive to PYD, no study has attempted to capture the dynamic relations between youth and their context. Given the importance of understanding PYD, this study aims to examine the construct and predictive validity of the Five Cs model of PYD in an Irish sample of adolescents. Not all person-context relations will result in PYD, therefore it is important to examine the validity of the construct of PYD across diverse settings, echoing calls from PYD researchers who have increasingly argued for evaluating diverse contexts for relational developmental systems that promote PYD.

### The “Five Cs” model of PYD

The Five Cs model of PYD emphasizes the strengths of adolescents. Framed by developmental systems theories, which place a strong focus on the plasticity (i.e., potential for systematic change) of development (Lerner, 2004), the model proposes that positive development occurs if the strengths of youth are aligned systematically with positive, growth promoting resources in the ecology of youth (i.e., “developmental assets,” Benson et al., 2006). The positive development that results from this alignment can be operationalized by “Five Cs”—Competence, Confidence, Connection, Character, and Caring (Eccles and Gootman, 2002; Roth and Brooks-Gunn, 2003; Lerner, 2004). Competence represents a positive view of one's actions in domain specific areas; Confidence is an indication of an internal sense of overall positive self-worth and self-efficacy and one's global self-regard; Connection refers to positive bonds with people and institutions; Character is an indication of an individual's respect for societal and cultural rules; and Caring is an indication of a person's sense of sympathy and empathy for others (Lerner et al., 2005). These domains are interactive and young people require healthy development in all of them (Dukakis et al., 2009). PYD is regarded as a linear combination of the Five Cs, whereby higher scores on each of the Cs contributes to the higher-order factor of PYD. Furthermore, it is hypothesized that when an adolescent manifests these Five Cs over time, they are more likely to be on a healthy life trajectory marked by contributions to self, family, community, and civil society (i.e., Contribution—the sixth C; Lerner, 2004), and less likely to be on a trajectory of risk and problem behavior (i.e., substance abuse, delinquency and depression; Jelicic et al., 2007). However, further research directly examining these correlates in non-North American populations is necessary to establish the utility and generalizability of theories of positive youth development.

### Previous research on the Five Cs model of PYD

Intensive empirical analysis has been conducted on the Five Cs Model through a number of studies (Lerner et al., 2005; Jelicic et al., 2007; Phelps et al., 2009; Bowers et al., 2010; Geldhof et al., 2013). These studies have demonstrated good internal consistency for each of the Five Cs (Lerner et al., 2005) and construct and predictive validity (Jelicic et al., 2007). However, despite the available evidence supporting the validity of the PYD measure, concerns remain. For instance, in terms of the structure of the Five Cs Model, previous findings suggest that some of the Cs may represent the same latent construct and that additional higher-order factors may exist (Lerner et al., 2005; Jelicic et al., 2007). Furthermore, revisions have been made to the indicators of constructs (Bowers et al., 2010). For example, the subscale of athletic competence was removed from the competence factor, and the subscale physical appearance was added to confidence (Bowers et al., 2010). More recently, research has used eight waves of longitudinal data to assess the structure of the Five Cs model of PYD (Geldhof et al., 2013), and found the structure of PYD to be notably different between younger and older adolescents. It is unknown whether these differences in structure between younger and older adolescents represent actual change in the conceptualization of positive development, or age-related measurement bias.

Furthermore, previous PYD research have drawn comparisons between males and females (with higher PYD observed in females; Lerner et al., 2005; Phelps et al., 2009), however, no analysis has been conducted to examine whether the underlying factors perform the same across gender (i.e., is the measurement model invariant between males and females). Previous literature has illustrated that adolescent perceptions of gender roles, for example masculinity, may inhibit emotional expression in males (e.g., empathy; O'Beaglaoich et al., 2013). Gender differences have also been consistently found regarding empathy and self-esteem (e.g., McMullin and Cairney, 2004). Gender differences in how indicators of PYD function for males and females have not been examined. Therefore, it is important to ascertain whether the factorial structure of the PYD measure is equivalent across gender (i.e., if the measure operates the same for males and females). Policy, assessment and intervention depend on the ability to compare factors across groups, therefore, it is imperative that the PYD measure reflects the phenomena under investigation, rather than measurement bias (Behl et al., 2001).

In conclusion, youth are likely to draw upon different internal and external resources within settings to promote positive development. In order to provide teachers and youth development practitioners with a tool to index positive development in young people, the measure of PYD must be applicable across settings. This study is the first to examine the structure and psychometric properties of a PYD measure in a European sample of adolescents. Extending the measurement of PYD to international samples is important given the increasing interest in using the PYD framework for youth program evaluation (e.g., Brady et al., 2011). Not all person-context relations will result in PYD, therefore it is important to examine the validity of the construct of PYD. In addition, there is a pressing need to examine the reliability and validity of existing measures of PYD across age and gender. The present study addressed these gaps by examining the psychometric properties of the Five Cs Model of PYD.

### The present study

Based on the above cited theory and research, several hypotheses were developed. First, the five-factor model of positive youth development (i.e., competence, confidence, connection, character, and caring) was expected to be a good fitting model for Irish adolescents. It was also hypothesized that the five-factor model would demonstrate equally good fit across gender, and across age group (i.e., early adolescence vs. late adolescence). In addition, PYD was hypothesized to be negatively related to both depression and risk-behaviors and positively related to contribution across males and females.

## Method

### Participants

A total sample of 672 respondents aged 11–19 (*M* age = 14.81, *SD* = 1.64, males = 57.6%) participated in the research. Participants were students attending 11 post-primary schools located in the Mid- and North-West region of the Republic of Ireland. Three schools were single sex (two all-male *n* = 123, one all-female *n* = 20), and eight schools were mixed-gender (*n* = 446), including one categorized as disadvantaged (*n* = 83). Students from across all stages of the Irish Post-Primary school system took part in the research, with 1st year (*n* = 146; 21.7%), 2nd year (*n* = 130; 19.3%), 3rd year (*n* = 86; 12.8%), 4th year (*n* = 110; 16.4%), 5th year (*n* = 184; 27.4%) and 6th year (*n* = 16; 2.4%) included. The majority of students were identified as “Born in Ireland” (*n* = 277, 41.2%), while 15.3% indicated other (e.g., England, US; *n* = 50, 7.4%). A large proportion of parents did not answer this question (*n* = 345, 51.3%). In terms of ethnicity, the majority were identified as “White” (*n* = 318, 47.3%), while a further 0.9% (*n* = 3) identified as “Mixed race” and “Asian” respectively, and less than 1% identified as “Other,” while a large proportion of parents did not complete this question (*n* = 345, 51.3%). Twenty-two percent of participants indicated living in an “Urban” area (*n* = 148), while 67.4% (*n* = 453) lived in rural areas, with missing data accounting for 10.6% (*n* = 71) of respondents. Mother's education was also reported, with a generally even distribution among different levels of education (8.3% Junior Certificate or less; 12.6% Leaving Certificate; 11.3% sub-degree; 7.4% primary degree; 7.9% professional qualification; 0.3% “Other.” 52.2% missing).

University ethical approval was obtained for this study and parent consent and student assent obtained before surveys were administered. After consent was obtained, the researcher administered instructions and surveys in class. All instruments were self-report measures, and the survey took approximately 35 min to complete.

### Measures

#### Positive youth development (PYD)

The Positive Youth Development “Five Cs” measure for grade 8–12 (Lerner et al., 2005; Phelps et al., 2009; Bowers et al., 2010) was used to assess PYD. This self-report measure consisted of a total of 15 subscales which serve as indicators of each of the Five Cs.

For *Competence*, subscales of the Self-Perception Profile for Children (SPPC; Harter, 1988) were used to represent academic, social and athletic competence (6 items per subscale), in addition to a single-item measuring school grades. A structured alternative response format (Harter, 1988) was used to assess perceived competence in each domain (excluding grades). Participants were asked to choose between two types of teenagers. Once they selected which person they are most like, they were asked to decide if it is “*really true for me*” or “*sort of true for me*.” For example, an academic competence item was “Some teenagers feel that they are pretty intelligent, BUT Other teenagers question if they are intelligent.” Items were counterbalanced, with each item scored from 1 to 4, with four reflecting higher perceived competence. The overall reliability of subscales in the current study is shown in **Table 3**.

*Confidence* was defined by a composite of two^1^ subscales: positive identity and self-worth (6 items each). The positive identity items were derived from the Profiles of Student Life-Attitudes and Behaviors Survey (PSL-AB; Benson et al., 1998), with a response format for items (e.g., “All in all, I am glad I am me”) ranging from 1 = *strongly disagree* to 5 = *strongly agree*. Self-worth was assessed using the subscale from the SPPC (Harter, 1988) with a structured alternative response format. Each item was scored from 1 to 4, with higher scores on subscales reflecting higher perceived confidence.

*Connection* consisted of three subscales of the PSL-AB (Benson et al., 1998) that measured connection to family (5 items), school (7 items), and community (5 items), and a subscale of the Teen Assessment Project Survey Question Bank (TAP; Small and Rodgers, 1995) to assess peer connection (4 items). The majority of items for the family, school and community (e.g., “In my neighborhood, there are lots of people who care about me”) subscales used a likert response format ranging from 1 = *strongly disagree* to 5 = *strongly agree*. A single item measuring connection to family (“If you had an important concern about drugs, alcohol, sex, or some other serious issue, would you talk to your parent(s) about it?”) used a likert format with responses ranging from 1 = *no* to 5 = *yes*. The items measuring connection to peers (e.g., “I trust my friends”) ranged from 1 = *never true* to 5 = *always true*. Higher scores were indicative of higher perceived connection.

The factor *Character* was defined by three subscales of the PSL-AB (Benson et al., 1998) that assessed social conscience (6 items), valuing of diversity (4 items) and personal values (5 items). A further subscale of the SPPC (Harter, 1988) measured behavioral conduct (6 items). For the subscales of personal values (e.g., “Telling the truth, even when it's not easy”) and social conscience (e.g., “Helping other people”), participants are asked to rate how important each item is in their lives, with response formats ranging from 1 = *not important* to 5 = *extremely important*. Three valuing of diversity items asked participants to think about the people who know them well and how they think they would rate them on each of the items (e.g., “Knowing a lot about people of other races”), using a response format ranging from 1 = *strongly disagree* to 4 = *strongly agree*. The behavioral conduct subscale was measured by using the Harter (1988) structured alternative response format. Higher scores indicated higher perceived character.

The fifth factor *Caring* comprises of five modified items from the Eisenberg Sympathy Scale (ESS; Eisenberg et al., 1996) and four items adapted from the Empathic Concern Subscale of the Interpersonal Reactivity Index (IRI; Davis, 1980), with a response format ranging from 1 = *not at all like me* to 5 = *very much like me*. High scores indicate higher levels of sympathy. In line with previous research (e.g., Phelps et al., 2009), individual items were randomly combined to form packets in order to enhance reliability (see **Table 3**). For the nine Caring items, the average of three sets of three items form packets 1, 2, and 3, respectively. All subscale item responses are rescaled to a 0–12 point scale.

#### Depression

Depression was measured by the 20-item Center for Epidemiological Studies Depression Scale (CES-D; Radloff, 1977). Using a likert response format ranging from 0 = *rarely or none of the time (less than 1 day)* to 3 = *most or all of the time (5–7 days)*, participants report how often they felt a particular way during the past week. Cronbach's alpha for the current sample was 0.88. Scores of greater than 16 are indicative of clinically significant depressive symptoms (Radloff, 1977).

#### Contribution

Contribution was measured using two equally weighted subscales of ideology and actions derived by Lerner et al. (2005). The ideology subscale (6 items) was obtained from the Teen Assessment Project Survey Question Bank (TAP; Small and Rodgers, 1995) and two items created by Lerner et al. (2005). This subscale assessed the importance of contribution to the individual's identity and future self. The items from TAP use a likert response scale ranging from 1 = s*trongly agree* to 5 = *strongly disagree*. The action subscale assessed leadership, service and helping (Lerner et al., 2005; e.g., “During the last 12 months, how many times have you been a leader in a group or organization?”) A likert response format was used, ranging from 1 = *never* to 5 = five *or more times*. The composite contribution scores were rescaled from 0 to 100, with higher scores illustrating greater levels of contribution. Internal reliability for grades 7–12 have been good, ranging from 0.75 to 0.81 (Geldhof et al., 2013), while in the current study Cronbach's alpha was also good (α = 0.76).

#### Risk behaviors

Risk behaviors were measured using scales of substance use and delinquency derived from the PSL-AB and the Monitoring the Future (2000) questionnaires. Four items were used to measure substance use or abuse. Participants were asked to indicate during the last 12 months whether they had done any of the following (e.g., smoking, using illegal drugs). The questions use a likert response format ranging from 1 = *never* to 5 = *regularly*. Four items were also used to measure delinquency. Participants were asked how many times during the last 12 months they have engaged in particular activities (e.g., “How many times have you hit or beat up someone?”). A likert response format was used, ranging from 1 = *never* to 5 = five *or more times*. All items were rescaled 0–5 and summed to form a composite measure of risk behaviors. Cronbach's alpha for the current study was 0.77.

### Statistical analyses

#### Missing data

Expectation maximization (EM) analysis was undertaken to impute missing data. EM is considered an excellent procedure for handling missing data (Allison, 2001). EM is advised when data are MCAR or MAR (Scheffer, 2002) and the percentage of missing data is, at most modest (i.e., less than 30%: Peugh and Enders, 2004). Little's Missing Completely At Random (MCAR) test was significant, $\chi_{\left(5112\right)}^{2}=5658.00$, *p* < 0.001, suggesting the data was not missing at random (MAR). It is likely that Little's MCAR test was significant due to an observed fatigue effect in the data, with higher rates of missing data observed at the end of the questionnaire than at the start. Thus, it can be assumed that the missing values carry no information regarding the missingness of other variables, and may be deemed MAR (Little and Rubin, 1987). In addition, as the highest level of “missingness” in the dataset was less than 30% (28.6%), the EM algorithm for imputing missing data was utilized.

#### Confirmatory factor analyses

In order to conduct a validation study of the proposed models, confirmatory factor models were specified and estimated using AMOS 20 (Arbuckle, 2011). Model parameters were estimated using maximum likelihood estimation. The best-fitting model was then subject to multi-group analysis where the factorial invariance of the model was tested across gender and young and older adolescent age groups.

Following guidelines from Byrne (2010), the adequacy of the fit between the specified Five Cs model and the observed data (i.e., model fit) was evaluated using a number of criteria; chi-square statistic; absolute fit was assessed by using the chi-square/*df* ratio (*Q*) and the Root Mean Square Error of Approximation (RMSEA) with 90% confidence intervals (90% Cl); and comparative fit was assessed using the Tucker-Lewis index (TLI), and Bentler's comparative fit index (CFI). Due to the sensitivity of the chi-square statistic to sample size, additional fit indices are also used as indicators of model fit. Rigorous thresholds were used to assess model fit: *Q* < 5, RMSEA ≤ 0.08, CFI and TLI ≥ 0.90 reflect adequate fit, while *Q* < 2, RMSEA ≤ 0.06, CFI and TLI ≥ 0.95 indicate excellent fit (Tabachnick and Fidell, 2007; Byrne, 2010). In addition, the standardized root-mean-square residual (SRMR) was also reported as recommended (Hu and Bentler, 1999), with values less than 0.08 indicative of acceptable model fit. When comparing the relative fit of two competing models, the Akaike Information Criteria (AIC) and delta AIC (Δ AIC; i.e., larger AIC minus smaller AIC) were used. The lower AIC value depicts the preferred model.

#### Invariance analysis

Multiple group analyses were used to assess the factor structure of the PYD model using CFA across age (early vs. late adolescence) and gender groups. Tests for invariant factorial structure of the specified models were conducted using multiple-group CFAs to fit a series of hierarchically nested factor structures (Chen et al., 2005). By assessing invariance, we can determine if individual factor subscales are functioning similarly across groups (e.g., gender, age).

First, the model is fitted to both groups separately to establish baseline model fit of the hypothesized model. Second, configural invariance is assessed by allowing the same set of subscales to form a factor in each group while allowing all model parameters to be freely estimated. If the configural invariance fits according to the model fit criteria outlined above (i.e., *Q* < 5, RMSEA = 0.08, CFI and TLI = 0.90), subsequent tests may be conducted. Metric (weak) invariance assesses the factor loadings across groups, first for first-order factor-loadings, then for second-order factor-loadings (Steenkamp and Baumgartner, 1998). Equivalence at the metric level allows the comparison of relationships. Scalar (strong) equivalence between groups is then tested by constraining factor loadings and intercepts to be equal. When both factor loadings and intercepts are invariant (i.e., scalar invariance), mean differences on the higher order latent factor (i.e., PYD) can be tested. Following Widaman and Reise (1997), the disturbances (i.e., error residuals) of the first-order factors were also tested.

Measurement invariance is supported when constrained models do not provide poorer fit as indicated by fit indices (i.e., ΔCFI) and the chi-square difference test. The chi-square difference test is deemed inappropriate in isolation, therefore the ΔCFI index with a cut-off criterion of <0.01 is used (Byrne, 2010). If a significant difference between groups was identified, modification indices and the factor-ratio method (Cheung and Rensvold, 2002) were utilized to identify group differences.

## Results

### Establishing the PYD model

A number of CFA models were specified based on previous model conceptualizations (Lerner et al., 2005; Phelps et al., 2009; Bowers et al., 2010). A test of the five-factor model of PYD (Lerner et al., 2005; Phelps et al., 2009) with no correlations between indicators failed to meet the recommended criteria for adequate model fit (see Model A, Table 1). Next, correlations were included between Harter subscales due to shared method variance (Phelps et al., 2009; Bowers et al., 2010), but the model again failed to meet the recommended criteria (Model B). However, all the lower-order and higher-order factor loadings were significant (range = 0.37-0.84) and above the minimum threshold of 0.30 (Bowers et al., 2010). This suggests that the structure of the Five Cs model was appropriate. Therefore, the five-factor model with higher order PYD factor was retained and subjected to model modification.

**Table 1:** **Confirmatory factor analysis of the five Cs measure of PYD (***N*** = 672)**.

**Model**	**χ^2^**	**df**	***Q***	**RMSEA**	**90% CI**	**SRMR**	**TLI**	**CFI**	**AIC Model**	**Δχ^2^**
(A)	611.26	90	6.79	0.09	0.09, 0.10	0.09	0.79	0.84	703.26	–
(B)	421.47	89	4.74	0.08	0.07, 0.08	0.06	0.86	0.90	515.47	189.797^***^
(C)	393.16	88	4.47	0.07	0.06, 0.07	0.06	0.87	0.91	489.16	28.31^***^
(D)	355.47	87	4.09	0.07	0.06, 0.07	0.06	0.89	0.92	453.47	37.69^***^

*Model (A), Five factor model with Bowers et al. (2010) correlations; Model (B), Residual errors of Caring and Character added; Model (C), Subscales of connection to family and behavioral conduct allowed covary; Model (D), Subscales of connection to peers and social competence allowed covary; 90% CI, 90% Confidence Interval; Δχ^2^, chi-square difference test; df, degrees of freedom; Q, absolute fit; RMSEA, root mean square error of approximation; SRMR, Standardized root-mean-square residual; TLI, Tucker-Lewis Index; CFI, Comparative Fit Index; AIC, Akaike Information Criteria*.

*** *p < 0.001*.

#### Modifications of the positive youth development measure

**Figure 1:**
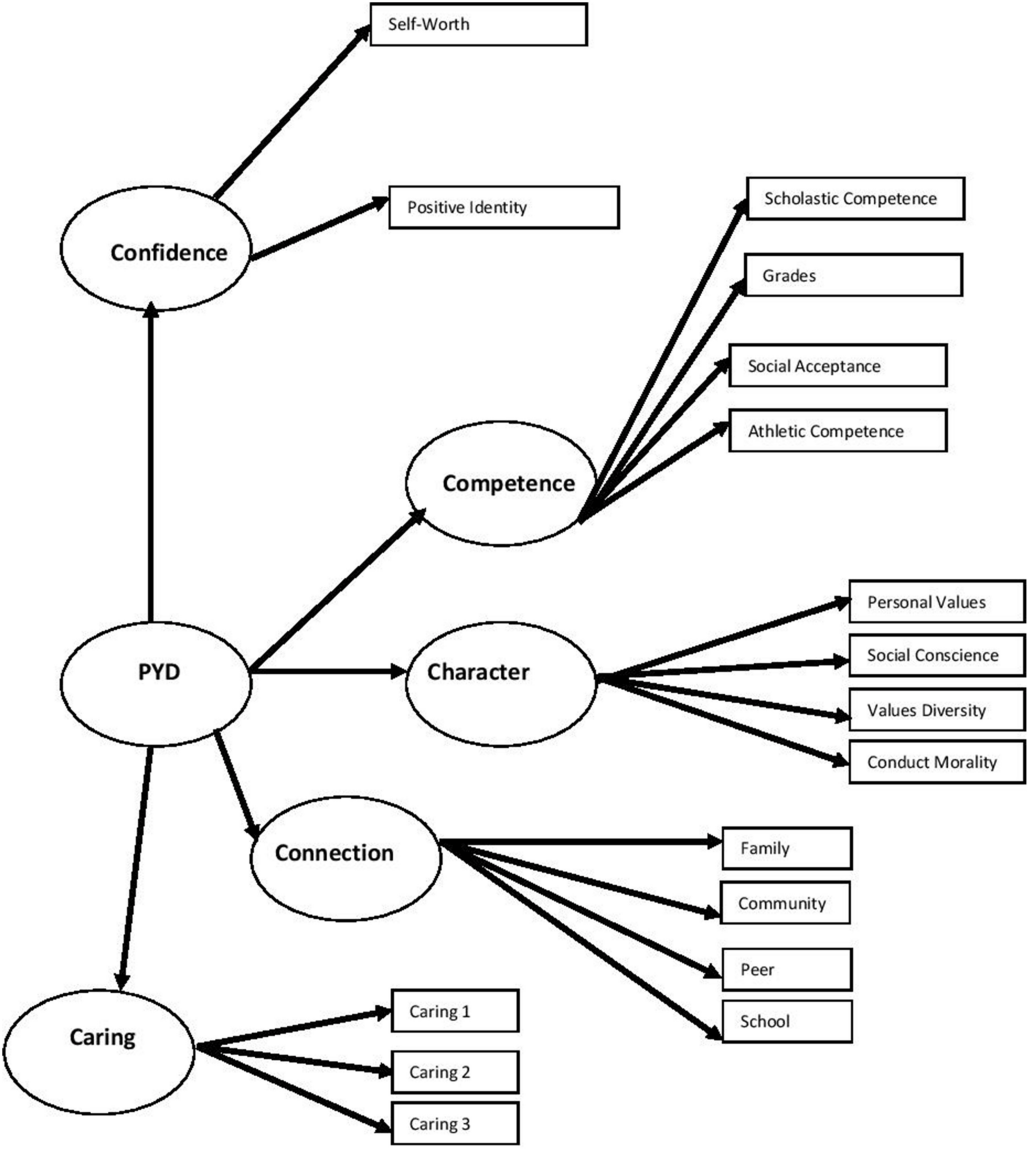
**Five Cs model of positive youth development**. PYD, positive youth development. Residual terms and covariances are omitted.

The content of item pairs with high modification indices was examined and models were re-specified if theoretical justification for the changes was established (Thompson, 2004). Reviewing the modification indices, a number of additional parameters were added. The inclusion of a covariance between Caring and Character is supported by previous research that included covariations between these two factors (e.g., Jelicic et al., 2007). The other covariances between connection to peers and social competence, and connection to family and behavioral conduct, were deemed theoretically appropriate given previous research suggesting relationships between social competence and ability to make friends (e.g., Gottman et al., 1975), and family relationships and adolescent morality (e.g., White, 2000). This model was a significantly improved model fit as assessed by the χ^2^ difference test, and displayed adequate fit to the data (see Model D, Table 1). Factor loadings for the final model can be seen in Table 2, while a graphical depiction of the model is shown in Figure 1.

**Table 2:** **Standardized and non-standardized factor loadings (and standard errors) for the five-factor model of PYD**.

**Item**	**β**	**B**	**SE**
**CARING**			
Caring 1	0.55	1.00	–
Caring 2	0.80	1.42	0.11
Caring 3	0.79	1.47	0.11
**CHARACTER**			
Social Conscience	0.81	1.00	–
Personal Values	0.68	0.76	0.05
Valuing of Diversity	0.67	0.93	0.06
Behavioral Conduct	0.35	0.42	0.05
**COMPETENCE**			
Social	0.64	1.00	–
Physical	0.45	0.91	0.16
Academic	0.52	0.94	0.16
**CONFIDENCE**			
Self-Worth	0.74	1.00	–
Positive Identity	0.70	0.66	0.05
**CONNECTION**			
Community	0.67	1.00	–
Peer Connection	0.51	0.65	0.06
Family Connection	0.62	0.86	0.06
School Connection	0.71	0.95	0.06
**PYD**			
Caring	0.34	0.47	0.07
Character	0.54	1.05	0.10
Competence	0.62	0.93	0.10
Confidence	0.81	1.29	0.11
Connection	0.94	1.86	0.12

*Factor loadings are all statistically significant (p < 0.001)*.

#### Reliability

The internal reliability for each individual subscale was acceptable (>0.70). Looking at the subscale reliability, the internal reliability of the PYD total scale (α = 0.72; 95% *CI* = 0.69–0.75), and majority of subscales (range α = 0.45[competence]-0.75[caring]), was good. The internal reliability of the Competence subscale was not satisfactory (α = 0.45, 95% CI = 0.37–0.52). However, in order to facilitate comparisons to previous research, the competence subscale was retained. Alpha coefficients and confidence intervals for all subscales (and validation measures), as well as means, standard deviations, and score ranges are presented in Table 3.

**Table 3:** **Descriptive Statistics for study variables**.

**Subscale**	**M**	**SD**	**Cronbach's Alpha (α)**	**95% CI**	**Possible Range**	**Attained Range**	**Skew**	**Kurtosis**
Caring	8.70	2.05	0.75	0.71–0.78	0–12	1.67–12.00	−0.54	−0.18
Character	7.96	1.77	0.71	0.67–0.74	0–12	1.93–11.85	−0.46	0.09
Competence	6.50	1.59	0.45	0.37–0.52	0–12	0.70–10.75	−0.11	−0.05
Confidence	7.61	1.97	0.54^a^	–	0–12	0–12.00	−0.22	0.33
Connection	8.36	1.74	0.73	0.69–0.76	0–12	1.06–12.00	−0.57	0.59
PYD	7.83	1.26	0.72	0.69–0.75	0–12	3.09–11.35	−0.18	0.21
Contribution	50.88	14.30	0.76	0.73–0.79	0–100	14.58–93.75	0.16	−0.04
Depression	14.74	9.51	0.89	0.89–0.91	0–60	0–53.00	1.18	1.49
Risk	0.65	0.52	0.77	0.75–0.80	0–5	0.07–4.69	2.83	12.87

a *Correlation coefficient; Cronbach's alpha does not make conceptual sense for two-item measures, hence, correlation coefficients were calculated (Streiner, 2003). CI, Confidence interval*.

#### Construct validity

Construct validity refers to the degree to which a particular measure relates to other measures, based on theoretically derived hypotheses regarding the constructs being investigated (Carmines and Zeller, 1979). Two types of construct validity were assessed; convergent validity and known-groups validity.

#### Convergent validity

Convergent validity is measured by testing the relationship of PYD to indices of positive and negative development (i.e., contribution and risky-behaviors). Pearson Product Moment correlations and hierarchical multiple regressions were used to evaluate the hypothesized relationships. A summary of all correlations are presented in Table 4. Pearson Product Moment correlations illustrated consistent patterns between the Five Cs, PYD, and outcomes of contribution and risky-behaviors. For contribution, all correlations were significant (*p* < 0.001), with low-moderate (Dancey and Reidy, 2007) positive correlations across PYD subscales (range *r* = 0.16–0.49). Similarly for risky-behaviors, all correlations were significant (*p* < 0.05), with low to moderate negative correlations across PYD subscales (range *r* = −0.13 to −0.37).

**Table 4:** **Summary of intercorrelations for subscales and Total PYD, Contribution, Depression and Risk Scale Scores**.

**Subscale**	**CR**	**CH**	**CP**	**CF**	**CN**	**PYD**	**Contribution**	**Depression**
**CARING (CR)**								
Character (CH)	0.56^***^							
Competence (CP)	0.05	0.19^***^						
Confidence (CF)	0.10^*^	0.30^***^	0.54^***^					
Connection (CN)	0.32^**^	0.49^***^	0.41^***^	0.54^***^				
PYD	0.61^***^	0.74^***^	0.60^***^	0.71^***^	0.79^***^			
Contribution	0.34^***^	0.49^***^	0.29^***^	0.16^***^	0.43^***^	0.49^***^		
Depression	−0.01	−0.12^*^	−0.43^***^	−0.58^***^	−0.46^***^	−0.45^***^	−0.09^*^	
Risk	−0.26^***^	−0.34^***^	−0.16^***^	−0.13^*^	−0.37^***^	−0.37^***^	−0.16^***^	0.16^***^

*PYD, positive youth development*.

* *p < 0.05*,

** *p < 0.01*,

*** *p < 0.001*.

Hierarchical multiple regressions were employed to evaluate how well PYD subscales predicted contribution and risky-behavior, after controlling for age and gender. Assumptions for regression analysis (e.g., normal distribution, autocorrelations among residuals) were tested with no violations identified. Age and gender were controlled for in step one, and the Five C subscales were entered in Step two.

##### Contribution

The overall model for contribution was significant, *F*_(7, 660)_ = 52.78, *p* < 0.001, *r*^2^ = 0.36, adj. *r*^2^ = 0.35. Age and gender were both significant predictors. Four out of the five PYD subscales were significant, with character, competence, confidence, and connection predicting contribution. Notably, confidence was significantly negatively related to contribution, while character, competence, and connection were positively related to contribution (see Table 5 for summary of model results).

**Table 5:** **Multiple Hierarchical Regressions for PYD subscale and total scores and positive and negative outcomes**.

**Predictors**	**Contribution**						**Risky-Behaviors**					
	**B**	**SE**	**β**	***r*^2^**	***Adj. r*^2^**	***F* change**	**B**	**SE**	**β**	***r*^2^**	***Adj. r*^2^**	***F* change**
Demographics				0.04	0.03	12.24^***^				0.08	0.08	30.22^***^
Age	0.98	0.28	0.11^**^				0.05	0.01	0.16^***^			
Gender	3.37	1.02	0.12^**^				−0.08	0.04	−0.08^*^			
PYD Subscales				0.36	0.35	66.58^***^				0.23	0.23	24.06^***^
Caring	0.15	0.28	0.02				−0.01	0.01	−0.05			
Character	3.01	0.34	0.37^***^				−0.05	0.01	−0.16^***^			
Competence	2.43	0.35	0.27^***^				−0.02	0.01	−0.06			
Confidence	−1.56	0.31	−0.21^***^				0.03	0.01	0.10^*^			
Connection	1.99	0.35	0.24^***^				−0.08	0.01	−0.28^***^			

*Adj. r^2^, adjusted r^2^*;

* *p < 0.05*;

** *p < 0.01*;

*** *p < 0.001*.

##### Risky-behaviors

The overall model for risky behaviors was also significant, *F*_(7, 660)_ = 27.31, *p* < 0.001, *r*^2^ = 0.23, adj. *r*^2^ = 0.22. Age and gender were both significant predictors. Three out of the five PYD subscales were significant, with character, confidence, and connection predicting risky-behaviors. Notably, confidence was significantly positively related to risky-behaviors, while character and connection were negatively related to risky-behaviors (see Table 5).

##### Known-groups validity

Known-groups validity describes the ability of a measure to discriminate across different groups (e.g., clinical and non-clinical samples; Cronbach and Meehl, 1955). Therefore group comparisons were conducted using independent *t*-tests to assess the relationships between high (i.e., scoring 16 and higher) and low depression (i.e., scoring < 16; Radloff, 1977) groups and the subscales of PYD and total PYD scale scores.

Independent *t*-tests showed PYD total scores and subscales of character, competence, confidence, and connection were significantly different across both groups, with the high depression group illustrating significantly lower character, competence, confidence and connection scores (see Table 6). The subscale of caring was not significantly different between low- and high-depression groups (*p* > 0.05). Effect sizes illustrated a small effect size for the character subscale, and a large effect size for the competence, confidence, connection, and PYD total scale scores.

**Table 6:** **Means, Standard Deviations, and ***T***-Tests for PYD scale scores and depression**.

**Measure**	**Low Depression**			**High Depression**						
	***N***	***M***	***SD***	***N***	***M***	***SD***	***df***	***t***	***p***	***d***
Caring	437	8.69	2.04	235	8.71	2.08	670.00	−0.06	0.952	0.01
Character	437	8.10	1.74	235	7.69	1.78	670.00	2.87	0.004^**^	0.23
Competence	437	6.91	1.45	235	5.76	1.57	670.00	9.50	<0.001^***^	0.76
Confidence	437	8.26	1.70	235	6.40	1.87	670.00	13.08	<0.001^***^	1.04
Connection	437	8.83	1.48	235	7.47	1.85	396.89^a^	9.69	<0.001^***^	0.81
PYD	437	8.26	1.15	235	7.20	1.22	670.00	10.01	<0.001^***^	0.89

a *Degrees of freedom adjusted due to significant Levene's test of homogeniety of variance*.

** *p < 0.01*;

*** *p < 0.001*.

*d, Cohen's d effect size*.

### Factorial invariance of positive youth development

#### Gender invariance

The Five Cs model of PYD was tested for factorial invariance across gender (Table 7) using multiple-group CFAs to fit a series of hierarchically nested factor structures (Chen et al., 2005). The baseline model fit was good for both males, $\chi_{\left(88\right)}^{2}=189.63$, *p* < 0.001; *Q* = 2.16; RMSEA = 0.064 (90% *CI* = 0.051–0.076); CFI = 0.92; TLI = 0.89; and females, $\chi_{\left(88\right)}^{2}=195.29$, *p* < 0.001; *Q* = 2.22; RMSEA = 0.066 (90% *CI* = 0.053–0.078); CFI = 0.92; TLI = 0.89. Next, configural invariance (i.e., all model parameters to be freely estimated) was examined in order to establish model fit across gender, and was confirmed; $\chi_{\left(177\right)}^{2}=385.31$, *p* < 0.001; *Q* = 2.18; RMSEA = 0.046 (90% *CI* = 0.039–0.052); CFI = 0.92; TLI = 0.89.

**Table 7:** **Tests of Five-factor PYD measure for factorial invariance by gender**.

**Model**		**χ^2^**	***df***	**Δχ^2^**	**Δ*df***	**RMSEA**	**RMSEA (90% CI)**	**CFI**	**AIC**
**GENDER FACTORIAL INVARIANCE**									
(1)	Configural model	385.31	177	–	–	0.046	0.039, 0.052	0.92	639.31
(2)	First-Order factor loadings invariant	397.17	188	11.86	11	0.044	0.038, 0.050	0.92	629.17
(3)	First- and second-order factor loadings invariant	407.19	193	10.02	5	0.045	0.039, 0.051	0.92	633.19
(4)	First- and second-order factor loadings and intercepts of measured variables invariant	658.46	209	251.27^***^	16	0.062	0.057, 0.067	0.82	852.46
(4a)	First- and second-order factor loadings and intercepts of measured variables invariant–8 intercepts freed	447.77	201	40.58^***^	8	0.047	0.041, 0.053	0.91	657.77
(5)	First- and second-order factor loadings, intercepts, and disturbances of first-order factors invariant	455.60	206	7.83	5	0.047	0.041, 0.052	0.91	655.60

*RMSEA, root mean squared error of approximation; CFI, Comparative Fit Index; CI, Confidence Interval*.

*** *p < 0.001*.

The third step was to test metric (weak) invariance by constraining first, the lower-order factor loadings (model 2), and then the higher- and lower-order factor loadings (model 3), across gender groups. This assesses whether the factor loadings (i.e., the relationship between the latent factors and their indicators), function similarly across groups. The results showed no significant differences between the configural model and model 2 and model 3, indicating first- and second-order factor loadings functioned equivalent across gender groups (i.e., metric invariance). Thus, the PYD subscale indicators function similarly for both males and female adolescents.

The fourth step involves testing scalar invariance (model 4). Scalar invariance is used to assess whether the intercepts (i.e., the level of scores) of the indicators used in the model are the same across groups. Results of model 4 indicated significant differences between males and females (see model 4, Table 7). Using the factor-ratio method (Cheung and Rensvold, 2002), eight intercepts were found to differ significantly across gender; athletic competence, peer connection, positive identity, social conscience, academic competence, caring1, caring2, and caring3 (*p*s < 0.001). Model 4a depicts the model fit indices this model with eight constraints freed. Partial scalar variance was observed in model 4a, as a significant chi-square difference test, but a non-significant difference in ΔCFI (ΔCFI < 0.01) indicated partial scalar invariance between males and females. This suggests that some gender differences in PYD may emerge due to gender bias in scoring for these eight items. However, although group differences were observed between intercepts, this need not preclude the usefulness of these items in measuring underlying constructs (Cooke et al., 2001).

Subsequently, the invariance of disturbances of first-order factors were tested (model 5). The chi-square difference test was non-significant (*p* > 0.05) and the ΔCFI was negligible (0.003). Therefore, no gender differences in the disturbances (i.e., the error terms) of the first-order factors were observed. Thus, measurement invariance was ascertained at the metric (weak) level, while partial invariance was observed at the scalar (strong) level allowing comparison of scores between males and females.

As metric and partial-scalar invariance were observed, differences between groups in the latent-factor means were assessed. Using the partial scalar invariant model, first-order latent mean differences were observed across all five PYD subscales, with females scored higher on caring (Females *M* = 9.58, *SD* = 1.72; Males *M* = 8.09, *SD* = 2.02), character (Females *M* = 8.30, *SD* = 1.66; Males *M* = 7.71, *SD* = 1.78), and connection (Females *M* = 8.57, *SD* = 1.79; Males *M* = 8.21, *SD* = 1.70), where males scored higher on confidence (Males *M* = 7.99, *SD* = 1.86; Females *M* = 7.23, *SD* = 1.98), and competence (Males *M* = 6.76, *SD* = 1.48; Females *M* = 6.17, *SD* = 1.66). Latent mean differences were also tested in relation to the higher order factor of PYD. No significant difference was found between females and males (*p* = 0.72).

#### Early and late adolescence

The Five Cs Model of PYD was also tested for factorial invariance across age groups (younger [11–15 years old] and older adolescents [16–18 years old]) to assess whether the PYD model functioned similarly across the adolescent period. The results illustrated metric and scalar invariance across age groups (see Table 8). The invariance of disturbances of first-order factors was subsequently tested (model 5), with no difference observed in the disturbances of the first-order factors. Therefore measurement invariance was ascertained at the metric (weak) level and scalar (strong) levels illustrating that the Five Cs model of PYD functions similarly for both younger and older adolescents.

**Table 8:** **Tests of Five-factor PYD measure for factorial invariance by age group**.

**Model**		**χ^2^**	***df***	**Δχ^2^**	**Δ*df***	**RMSEA**	**RMSEA (90% CI)**	**CFI**	**AIC**
**AGE GROUP FACTORIAL INVARIANCE**									
(1)	Configural	401.03	177	–	–	0.050	0.044, 0.057	0.91	661.03
(2)	First-Order factor loadings invariant	419.46	188	18.43	11	0.049	0.043, 0.056	0.91	657.46
(3)	First- and second-order factor loadings invariant	424.78	193	5.32	5	0.049	0.043, 0.055	0.91	652.78
(4)	First- and second-order factor loadings and intercepts of measured variables invariant	464.33	209	39.55^***^	16	0.049	0.043, 0.055	0.90	660.33
(5)	First- and second-order factor loadings, intercepts, and disturbances of first-order factors invariant	475.40	214	11.07^*^	5	0.049	0.043, 0.055	0.90	661.40

*RMSEA, root mean squared error of approximation; CFI, Comparative Fit Index; CI, Confidence Interval*.

*** *p < 0.001*;

* *p < 0.05*.

As metric and scalar invariance were observed, differences in latent factor means could be assessed. Using the scalar invariance model, a significant difference was found for caring, character and connection, with younger adolescents scoring significantly higher on caring (Younger *M* = 9.00, *SD* = 2.02; Older *M* = 8.61, *SD* = 2.04), character (Younger *M* = 8.29, *SD* = 1.79; Older *M* = 7.70, *SD* = 1.63), and connection subscales (Younger *M* = 8.62, *SD* = 1.80; Older *M* = 8.15, *SD* = 1.71). No differences were found between age groups for competence and confidence factors (*p*'s > 0.05). Latent mean differences were also tested in relation to the higher order factor of PYD. A significant difference was found (Est = 0.32, *z* = 3.28, *p* = 0.001), indicating that younger adolescents (*M* = 8.09, *SD* = 1.27) had a significantly higher score on the PYD factor than older adolescents (*M* = 7.66, *SD* = 1.20).

## Discussion

The purpose of this study was to investigate the dimensionality, reliability and the validity of the Five Cs model of PYD with a sample of Irish adolescents. Confirmatory factor analyses indicated that in line with previous research (e.g., Lerner et al., 2005), the addition of a number of covariances (i.e., between caring and character; connection to peers and social competence; and connection to family and behavioral conduct) resulted in the Five C's model illustrating an adequate fit to the data (i.e., *Q* < 5; RMSEA < 0.08; CFI > 0.90). The current study also assessed the reliability of the Five Cs model of PYD. In line with previous research (Phelps et al., 2009; Bowers et al., 2010), the total and subscale scores evinced good scale score internal reliability. One exception to this was the competence subscale which illustrated poor internal reliability (α = 0.45; 95% *CI* = 0.37–0.52). This suggests that scoring on the subscale indicators of social competence, academic competence, and athletic competence did not display consistent inter-item scoring patterns. However, all indicators loaded significantly onto the latent factor of competence (i.e., social competence = 0.64; athletic competence = 0.45; academic competence = 0.52), supporting their inclusion in the model. Furthermore, low internal reliability is often found in scales with a low number of items (Nunnally and Bernstein, 1994), and a high value is not expected when measuring diverse aspects of an overarching construct such as athletic and academic competence (Sijtsma, 2009). Therefore the competence factor was retained.

In terms of construct validity, the PYD subscales showed good convergent and known-groups validity. Specifically, higher character, competence, and connection predicted higher contribution, while higher character and connection predicted lower risky-behaviors. In addition, PYD subscales of character, competence, confidence, and connection were significantly different across groups of high and low depression. Notably, the observed relationships between confidence and measures of contribution and risky-behaviors were not in the expected direction and contrasted previous research (Lerner et al., 2005). In particular, higher scores on the confidence subscale were related to lower contribution and higher risky-behaviors. This finding may be linked to two lines of thought. First, previous research that has associated overconfidence with narcissism (Morf and Rhodewalt, 2001; Landazabal, 2006). Narcissism has been associated with positive characteristics such as authority/leadership, assertiveness, and confidence, and negative characteristics such as a sense of entitlement, strong desire to be the center of attention, and willingness to exploit others (Raskin and Terry, 1988; Barry et al., 2007). In terms of outcomes, narcissism has been associated with conduct problems and internalizing problems in young people (Barry et al., 2003; Washburn et al., 2004). This is in line with the current study, where a positive association between confidence and risky-behaviors was observed. Future, research is needed to examine whether the confidence construct measured in the PYD model is in any way analogous to narcissism, and how different forms of confidence and narcissism relate to other indicators of positive and negative development. A second line of inquiry may also investigate the influence of cross-cultural differences in the way in which the confidence items are interpreted. Such differences in interpretation may account for the different scoring patterns. For example, the confidence item “when I am an adult, I'm sure I will have a good life,” may elicit connotations of the recent economic recession in Ireland and the widely reported loss/lack of employment throughout the country. Ireland's adult employment rate (59%) is lower than the Organization for Economic Co-operation and Development (OECD) average (65%) and the adult employment rate in the US (67%; Better Life Index, 2013). Thus, within the context of the economic recession, Irish adolescents may interpret and answer this confidence item differently compared to adolescents in the US. Future research is therefore needed to clarify the interpretation of the confidence items across cultures.

In terms of measurement invariance (i.e., assessing whether the PYD scale performed consistently across gender and age), metric and scalar invariance were observed across age groups, suggesting that the PYD measure functions similarly across younger and older adolescents. This supports previous research that illustrated measurement invariance of the Five Cs model over an 8-year longitudinal study (Geldhof et al., 2013), and illustrates that the Five Cs model is a conceptually valid framework of positive functioning across adolescence.

While previous research examined the age invariance of the Five Cs model of PYD (e.g., Geldhof et al., 2013), no research has assessed the functioning of the Five Cs model across gender. The current study observed metric and partial scalar invariance across males and females. This indicates that, while the indicators of each of the “Cs” function the same for males and females (i.e., metric invariance), a number of indicators differed in terms of the level of scoring. Most notably, all indicators of the Caring factor were found to differ across gender. This suggests that differences in mean caring scores between genders may be biased due to males scoring on a lower range of scores. The result of this is that males and females may be equally empathetic, but females may score higher on the current measure due to gender bias on a number of items. The finding of invariance at the metric (weak) level assures that comparisons can be made for the caring subscale as to the relationships between the factors (i.e., factor coefficients) across groups (Clench-Aas et al., 2011). Caution however, should be exercised in interpreting analyses involving comparison of latent means of caring between groups (Clench-Aas et al., 2011). On the other hand, the observed gender differences are in line with previous research showing females score higher on kindness (Linley et al., 2007) and empathy (Litvack-Miller et al., 1997; McMullin and Cairney, 2004).

Latent mean scores of PYD and the Five Cs were also assessed, and indicated a number of differences across groups. For instance, results indicated that females scored higher on the factors of caring, character and connection, while males scored higher on the factors of confidence and competence. This suggests that PYD may not manifest in a uniform manner across gender groups. These discrete differences between males and females on each of the Five Cs are in contrast to previous research that highlighted females scoring consistently higher on all Five Cs (Lerner et al., 2008). However, the finding that females scored higher on caring, character, and connection, and lower on competence and confidence compared to males, is in line with previous research illustrating significant gender typing by adhering to gender-role standards of behavior (McMullin and Cairney, 2004; Linley et al., 2007). Latent mean differences were also assessed across age groups. Younger adolescents were found to score higher on caring, character, connection, and overall PYD scores. This indicates that PYD appears to decline from younger adolescence to older adolescence. These results concur with the findings of Harter (1998), who reported that many of the domains of positive self-concept decrease over the early adolescence years. Thus, the current findings were consistent with predicted developmental outcomes.

In sum, the Five Cs model of PYD was found to be an adequate structural model to depict positive functioning in Irish adolescence. In general, the PYD subscales were related to measures of contribution and risky-behaviors in line with theoretically derived hypotheses, and the PYD subscales (with the exception of caring) were able to discriminate non-clinical (i.e., scores less than 16) and clinically significant depression scores (i.e., scores above 16). The Five Cs measure was also found to be a robust measure across younger and older adolescent age groups. Notably, a number of gender differences were observed, indicating that PYD may manifest differently across gender groups.

### Limitations

A number of limitations and concerns should be addressed. First, questions remain over the structure of the model. The modifications of the PYD model, including the shared variance between the first-order factors of caring and character, suggest that some of the C's may represent the same latent construct. Thus, researchers need to continue to take care to examine the measurement properties of their PYD scale in each study where the measure is being used.

Additionally, the caring factor of PYD was consistent in its failure to significantly predict either contribution or risky-behaviors, and differentiate between groups of individuals with high and low depression scores. Given the low factor loading onto the PYD construct, and the shared variance with the character construct, future research may look at the items used to assess caring in the model of PYD. Previous iterations of the Five Cs model have already changed the items used in the caring construct, changing the subscale from sympathy to empathy (Lerner et al., 2005; Phelps et al., 2009). The addition of further items assessing constructs related to empathy, and fitting within the definition of caring, may strengthen the caring factor. For example, the measurement of compassion is defined as being open to and moved by the suffering of others, desiring to ease suffering of others, and offering others patience and non-judgemental understanding (Neff, 2003). While related to empathy (i.e., moved by the suffering of others), the measurement of compassion assesses additional individual motivations (e.g., offering non-judgemental understanding). Thus, the addition of compassion may strengthen the measurement of caring within a PYD framework. Further research is required to assess whether the current findings are replicated in order to support additional changes to the caring construct.

In conclusion, the present study provides support for the structure of the PYD model in an Irish context. In addition, the reliability and validity of the Five Cs Model of PYD is supported as the theoretically expected relations with positive (contribution) and negative (depression and risky-behavior) indicators of development were generally observed. Therefore, the Five Cs model of PYD provides practitioners, teachers and youth leaders, with both a common vocabulary to discuss healthy development, and a tool to measure PYD among adolescents. However, further research is needed to clarify the gender differences in a number of indicators. Notably, the results suggest that PYD is not a homogeneous construct for both males and females. Further, work is necessary then to elucidate the underlying factors of PYD that could potentially inform youth programs for both gender groups. Overall, the present findings suggest that the Five Cs model of PYD is a suitable model of positive functioning among adolescents in Ireland, and that this measure is useful and valid in relation to understanding expected relationships with positive and negative developmental indices.

## Author contributions

RC conceived of the study, while RC, CH, and MH participated in the study design and coordination, interpretation of the data, and drafted the manuscript. All authors read and approved the final manuscript and agree to be accountable for all aspects of the work in ensuring that questions related to the accuracy or integrity of any part of the work are appropriately investigated and resolved.

### Conflict of interest statement

The authors declare that the research was conducted in the absence of any commercial or financial relationships that could be construed as a potential conflict of interest.

## Footnotes

^1^An independent sample (*N* = 327) was used to compare the Phelps et al. (2009) PYD measure, and the measure outlined by Bowers et al. (2010) where the subscale of physical appearance was added as a subscale of confidence, and the subscale of athletic competence removed as a subscale of competence. The CFA of the Phelps et al. (2009) model resulted in a better model fit, $\chi_{\left(87,\;n=327\right)}^{2}=260.26$, *p* < 0.001, *Q* = 2.99, CFI = 0.90, TLI = 0.86, RMSEA = 0.074 (90% CI = 0.068–0.080), SRMR = 0.07, AIC = 358.26, compared to the Bowers et al. (2010) model, $\chi_{\left(87,\;n=327\right)}^{2}=280.23$, *p* < 0.001, *Q* = 3.22, CFI = 0.89, TLI = 0.86, RMSEA = 0.082 (90% CI = 0.071-0.093), SRMR = 0.08, AIC = 376.23. The Phelps et al. (2009) model of PYD was therefore retained and used in the current analyses.
